# Supplementary figures and images for: Estimated seroprevalence of SARS-CoV-2 antibodies among adults in Orange County, California
Source: Sci Rep. 2021 Feb 4;11:3081. doi: 10.1038/s41598-021-82662-x (PMC7862219; doi:10.1038/s41598-021-82662-x)

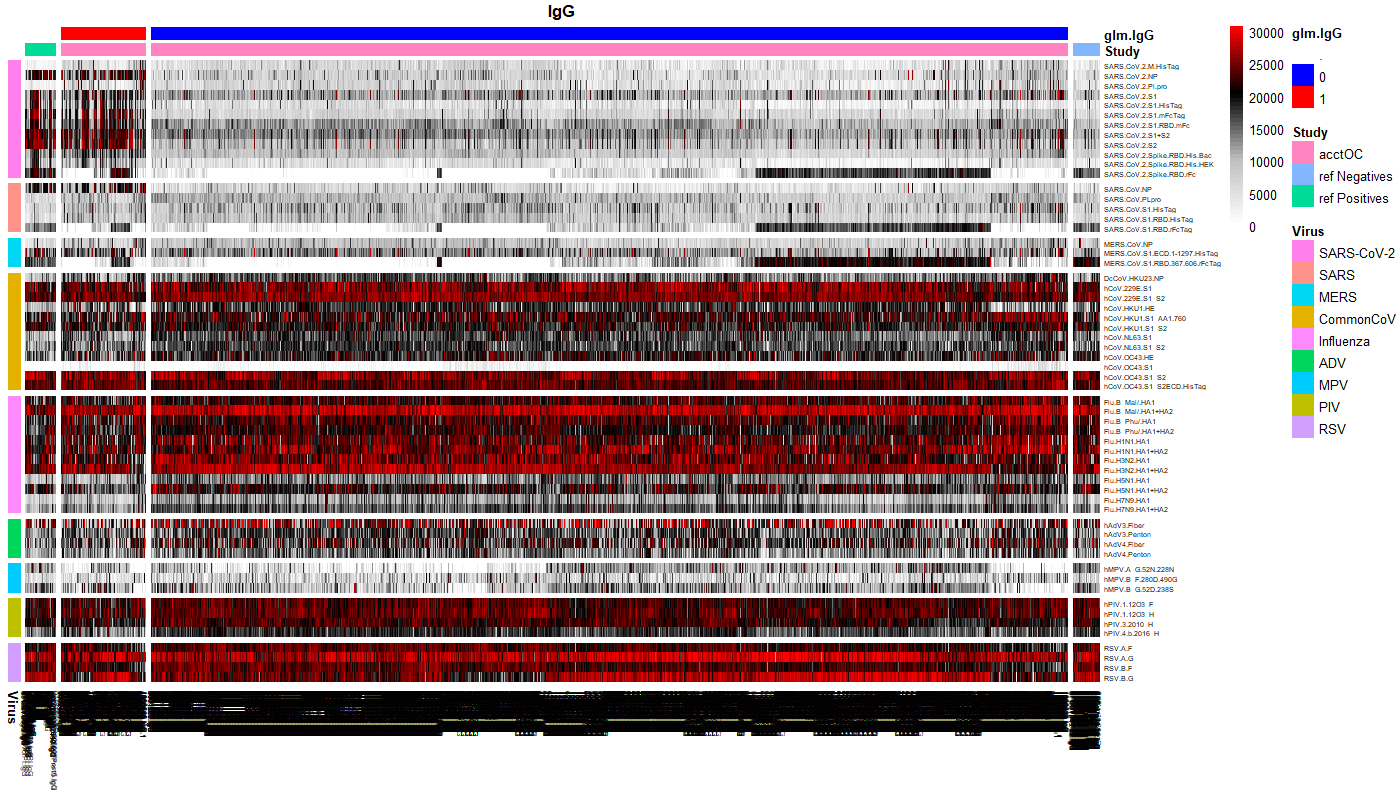

Supplement: Supplementary file 1 — Supplementary Information 1 [file 41598_2021_82662_MOESM1_ESM.png]
